# Reinstitution of pegvaliase therapy during lactation

**DOI:** 10.1016/j.ymgmr.2022.100938

**Published:** 2022-11-17

**Authors:** Frances Rohr, Ann Wessel, Cary O. Harding, Susan E. Waisbren, Krista Viau, Amy Kritzer

**Affiliations:** a Met Ed Co, Boulder, CO, USA; b Department of Genetics and Genomics, Boston Children's Hospital, Boston, MA, USA; c Department of Nutrition, Boston Children's Hospital, Boston, MA, USA; d Molecular and Medical Genetics, Oregon Health & Science University, Portland, OR, USA; e Harvard Medical School, Boston, MA, USA

**Keywords:** PKU, Phenylketonuria, Maternal PKU, Breastfeeding, Lactation, Pegvaliase, Phenylalanine ammonia lyase

## Abstract

Pegvaliase, an injectable form of phenylalanine ammonia lyase, is an enzyme substitution therapy for adults with phenylketonuria (PKU). Experience with pegvaliase during lactation is scarce. Limited evidence suggests that pegvaliase does not pass into breast milk. The case presented here describes the pregnancy and lactation experience of a woman with PKU who was treated with pegvaliase prior to pregnancy, discontinued pegvaliase and was treated with a phenylalanine-restricted diet in preparation for and during pregnancy, and then reinstituted pegvaliase two weeks after giving birth and throughout lactation. No pegvaliase activity was detected in pumped breast milk samples prior to reinstituting pegvaliase, and at doses of 80, 110 and 140 mg/week during lactation. The phenylalanine content of breast milk samples collected during pegvaliase therapy were not significantly different than controls, and the infant has grown and developed normally, indicating that pegvaliase therapy during lactation is safe.

## 1 Introduction

Phenylketonuria (PKU) is an inherited metabolic disorder due to phenylalanine hydroxylase (PAH) deficiency that results in elevated blood phenylalanine (Phe) concentrations [1]. If blood Phe is not controlled from infancy within the target range of 120–360 μmol/L [2] then patients are at risk of developmental disability, seizures and eczema. The traditional treatment for PKU is a Phe-restricted diet, however, this diet is difficult to follow and often results in suboptimal blood Phe [3] and adverse neuropsychological outcomes [4]. Pegvaliase pqpz^2^ (pegvaliase) is an injectable enzyme substitution therapy containing phenylalanine ammonia lyase that converts phenylalanine to trans-cinnamic acid and ammonia and can replace a Phe-restricted diet in those with PKU who respond to this treatment [5].

Published experience with pegvaliase during pregnancy is limited to three cases, all of whom were being treated with pegvaliase when the pregnancies came to attention [[6], [7]]. In one case, pegvaliase was discontinued and the pregnancy was subsequently miscarried at 7 weeks gestation [7]. In both other cases, the women continued pegvaliase after they and their metabolic teams considered the well-known risk to the fetus of poorly treated maternal PKU [[8], [9]] to be greater than the possible risk of pegvaliase therapy. Of the patients who remained on pegvaliase, in one case the pregnancy resulted in a miscarriage at 7 weeks gestation (50 weeks on pegvaliase therapy) [7] and in the second, the pregnant woman was induced at 37 weeks due to maternal hypertension with vaginal delivery of a healthy male infant [6]. However, this woman did not choose to breastfeed her infant; thus, there are no human data available regarding lactation during pegvaliase therapy. Additionally, there are no studies in humans regarding pegvaliase use during lactation. According to the prescribing information for the drug, pegvaliase was detected in maternal milk of pegvaliase-treated lactating non-hyperphenylalaninemic rats but not in their nursing offspring, and pegvaliase administration at 2.8 times the maximum human dose during lactation resulted in decreased pup weight and survival. While not contraindicated on the label, the pegvaliase prescribing information states that it may cause low Phe content in human milk and clinical judgement regarding pegvaliase use during lactation is recommended (FDA).

## 2 Case report

The history of this woman's PKU therapy has been previously reported [10]. Briefly, she was identified through NBS, has a severe PAH variant (R408W; IVS 7 nt3 G > C), and was treated with a Phe-restricted diet since infancy. She previously did not respond to sapropterin therapy, and she participated in the pegvaliase clinical trial. At the time she planned her first pregnancy, she was being treated with pegvaliase 60 mg daily. She discontinued pegvaliase therapy and re-instituted a Phe-restricted over a 12-week period. She maintained blood Phe in the recommended range during pregnancy and delivered a healthy female infant at 39 weeks gestation. The woman remained on a Phe-restricted diet while breastfeeding for 7 months but had difficulty maintaining the diet during lactation (blood Phe 1406 μmol/L at weaning). After weaning, she resumed pegvaliase therapy on an accelerated schedule, reaching efficacy at 6 weeks on 20 mg/day and increasing intact protein intake to 80 g/day. One sample of pumped breastmilk when the woman was treated with pegvaliase (20 mg/d or 140 mg/week) was tested and found to have no pegvaliase activity.

A second pregnancy was planned when her first child was 20 months old. At the time, she was on pegvaliase therapy (140 mg/week). The transition from pegvaliase to a Phe-restricted diet was accomplished over a 5-week period (Table 1); however, blood Phe was not in the recommended treatment range (<360 μmol/L) until 6 weeks after the pegvaliase was fully discontinued, and 13 weeks after that time, pregnancy occurred. During pregnancy, mean blood Phe was 271 μmol/L (range 55–752 μmol/L,) and mean blood tyrosine was 39 μmol/L (range 29–122 μmol/L). Medical food provided 75 g/d of protein until pregnancy occurred and then at least 90 g/d throughout pregnancy (Table 2).

**Table 1:** Plan for transitioning off pegvaliase and onto phe-restricted diet for second pregnancy.

Week	Palynziq Dose (mg/week)	Medical Food Protein (g/d)	Intact Protein (g/d)	Diet Description
1	140	35	40	No meat, eggs, dairy but continue higher protein grains; supplement with whey protein
2	80	35	40	No meat, eggs, dairy but continue higher protein grains; supplement with whey protein
3	40	55	20	No meat, eggs, dairy but continue some higher protein grains
4	20	75	5–6	Fruits, vegetables, low protein foods (250–300 mg PHE)
5	None	75	5–6	Fruits, vegetables, low protein foods (250–300 mg PHE)

**Table 2:** Mean blood phenylalanine and medical food and phenylalanine intake before during pregnancy.

Time period	Blood Phenylalanine (μmol/L) (number of samples)	Medical Food Protein (g/day)	Phenylalanine (mg/day)
3 months pre-pregnancy	439 (14)	75	275
1st trimester	310 (11)	90	420
2nd trimester	268 (19)	90	520
3rd trimester	235 (10)	90	915

The pregnancy was complicated by gestational diabetes requiring insulin from 24 weeks gestation on. A male infant was delivered vaginally at 39 weeks gestation (weight 3.8 kg, 70th percentile for age; length 52 cm, 77th percentile, head circumference 35.6 cm, 38th percentile [11]. The infant was breast-fed exclusively.

**Fig. 1:**
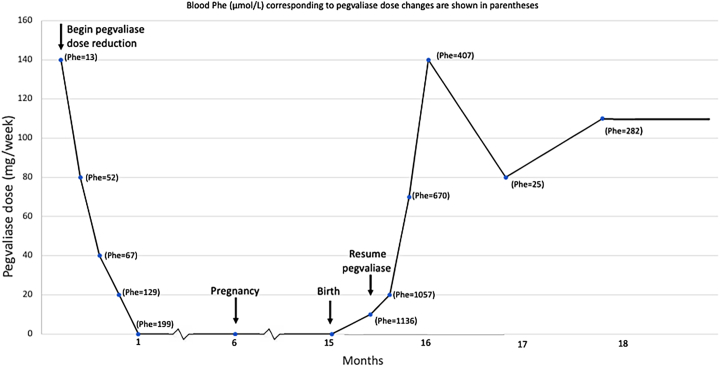
Pegvaliase dose before and during pregnancy and during lactation in a woman with PKU.

The woman remained on a Phe-restricted diet for 2 weeks after the birth of her son, then she resumed pegvaliase therapy. Her pegvaliase dose was escalated without adverse reactions as follows: 2.5 mg twice a week for Week 1, 10 mg twice a week for Week 2, 10 mg daily for Week 3 and 20 mg daily for Week 4 (Fig. 1). During the first 3 weeks, she remained on the same diet as during her third trimester, which consisted of 75 g of protein from medical food and approximately 15–20 g of protein from food. By the fourth week of pegvaliase, she resumed an unrestricted diet containing no medical food and at least 75 g of protein/day from intact protein, and, soon after that, 90–100 g protein/day. Once a normal diet was established, diet intake was recorded periodically.

Her blood Phe was below the physiologically normal range twice (26 and 25 μmol/L at 8 and 9 weeks post-partum), and, in response, the pegvaliase dose was decreased from 140 mg to 80 mg/week. Then, blood Phe rose to 282 and 264 μmol/L; therefore, the dose was increased slightly to 110 mg/week where it has since remained (Table 3). Diet intake was estimated in the post-partum period, using a simplified diet approach [12].

**Table 3:** Mean blood phenylalanine, pegvaliase dose, pegvaliase activity, and protein intake during lactation.

Lactation (Day)	Blood Phe (μmol/L)	Pegvaliase Dose (mg/week)	Breast Milk Pegvaliase Activity	Medical Food Protein (g/day)	Intact Protein (g/day)
1 (Birth)		0		90	15–20
9 (Breast milk sample)		0	nd		
12		10			
14	1136				
20		20			
23	1057				
27		70		0	75–90
31	670				
34		140			
37	407				
46 (Breast milk sample)		140	nd		
50	26			0	90–100
55		80			
57	25				
61 (Breast milk sample)		80	nd		
64	282				
86	264				
97		110			
119					
123 (Breast milk sample)		110	nd		
133	143				

nd = not detected.

The infant gained weight normally and met developmental milestones. He now consumes a normal diet for age, including one to two breast feedings daily. At his one-year checkup, growth percentiles were age-appropriate (weight 60th, length 64th, and head circumference 31st percentile) [11].

Developmental testing at age 14 months confirmed typical developmental progress. On the tests administered, scores between 85 and 115 indicate average performance (normative mean = 100, standard deviation = 15). On the Bayley Scales of Infant and Toddler Development, Third Edition, this little boy received a score of 110 on the Cognitive Scale, indicating abilities at the 16-month level. He attained a score of 100 on the Language Scale, with skills at the 15-month level in Receptive Communication and at the 12-month level in Expressive Communication. On the Bayley Motor Scale, he attained a score of 100, with fine motor skills at the 15-month level and gross motor skills at the 14-month level. The Receptive-Expressive Emergent Language Test-Third Edition (REEL-3) provides a more in-depth assessment of language. On this test, he received a score of 97 on the Receptive Language Scale (13-month level) and 94 on the Expressive Language Scale (12-month level), with an overall Language Ability Score of 95. His mother provided ratings for the Vineland Adaptive Behavior Scale, Third Edition. He received the following domain scores: 96 in Communication, 95 in Daily Living Skills, 97 in Socialization and 105 in Motor Skills. Overall, he demonstrated relative strengths in fine motor skills and relative weaknesses in early expressive language, although overall his performance was well within the average range.

Four breast milk samples were tested representing times during lactation when the woman was receiving no pegvaliase, her highest dose, her lowest dose, and her current dose (Table 3). Skim breast milk was evaluated for the presence of pegvaliase activity using a spectrophotometric method to detect the conversion of *L*-phenylalanine to *trans*-cinnamic acid as previously described [10]. No pegvaliase activity was detected in any breast milk samples from the patient (Table 3).

Total amino acid content of the breast milk samples was also evaluated to determine whether pegvaliase treatment had altered the phenylalanine content of the breast milk. Skim breast milk samples (2.5 mg total protein) were hydrolyzed in 1 mL 6 N hydrochloric acid at 100 °C for 24 h under vacuum. The hydrolysates were neutralized with sodium hydroxide and spiked with a known quantity of norvaline as an internal recovery standard before amino acid analysis by precolumn derivatization with 6-aminoquinolyl-N-hydroxysuccinimidyl carbamate (AQC, Waters AccQ Tag™), then separation by reverse phase ultra-high performance liquid chromatography and UV absorbance detection (Waters Acquity™ UPLC) using the Waters Masstrak Amino Acid Analysis method. The phenylalanine content of three breast milk samples taken while the mother was treated with pegvaliase was not different from that of the milk obtained before she reinitiated pegvaliase therapy or in comparison to a breast milk sample from an unrelated non-PKU woman collected at 36 days after parturition (Table 4).

**Table 4:** Amino acid content of breast milk hydrolysates.

Amino acid content (mg/100 mL, mean ± SEM)	Control milk samples* (*n* = 3)	Milk samples on pegvaliase (Days 46, 61, and 123)	*P* value (Students' *t-*test)
Phenylalanine	50.8 ± 12.7	27.2 ± 3.2	0.0722
Tyrosine	44.4 ± 10.8	24.2 ± 2.0	0.1398
Amino acid ratios			
Phenylalanine/proline	0.40 ± 0.02	0.38 ± 0.02	0.3361
Phenylalanine/alanine	1.24 ± 0.09	1.14 ± 0.07	0.2267
Phenylalanine/tyrosine	1.14 ± 0.02	1.12 ± 0.04	0.3182

Control milk samples included a sample collected on day 9 after birth from the mother in this report (and analyzed twice) and a sample from a non-PKU woman taken at 36 days after birth.

Compared to the first lactation experience, the woman described her experience as much more positive while on pegvaliase. While her goal was to breastfeed both of her children for one year, she had to make the difficult decision to stop breastfeeding her daughter early to resume pegvaliase therapy in order to attain metabolic control. Conversely, resuming pegvaliase soon after her son's birth enabled her to establish breastfeeding and continue until 15 months. It allowed her son to wean when he was ready as opposed to stopping for her own health benefit. She was able to focus on typical postpartum recovery and maintaining successful breastfeeding and milk supply as opposed to juggling medical food intake and tracking dietary intake while transitioning to the many demands of being a mother.

## 3 Discussion

Previous evidence suggested that it is unlikely that pegvaliase passes into breastmilk [[10], [13]], and this case further supports the lack of pegvaliase in the milk of a woman with PKU treated with pegvaliase. The most abundant proteins in breast milk, including casein and alpha-lactalbumin, are synthesized from individual amino acids in the mammary alveolar cell endoplasmic reticulum, trafficked to the alveolar cell membrane via the Golgi pathway and secreted via exocytosis into the alveolar duct [14]. Some maternal serum proteins such as immunoglobulins including IgA are selectively taken up from maternal circulation via specific transporters into the alveolar cells and then trafficked across the cell via transcytosis to be delivered to maturing milk in the duct. Minor amounts of other maternal serum components may enter milk via a paracellular pathway along with maternal leucocytes. Were pegvaliase, a large multimeric protein that has been heavily coated with polyethylene glycol (PEG), to enter breast milk from maternal circulation, this would most likely occur via the paracellular pathway only and so should result in only miniscule amounts of pegvaliase present in breast milk. In fact, we have not detected any pegvaliase activity in multiple breast milk samples from this single individual treated with pegvaliase at up to 180 mg pegvaliase per week. We have not utilized any antigenic methods to examine the milk samples for the presence of inactive pegvaliase protein or protein fragments, nor have we examined the fat component of the breast milk. Even if we had measured appreciable pegvaliase activity in the milk, pegvaliase is capable of metabolizing only free phenylalanine.

Phenylalanine content in breast milk is primarily provided by intact protein, mainly casein and alpha-lactalbumin; phenylalanine present in the primary amino acid sequence of these proteins would not be a substrate for pegvaliase. For this reason, despite the warning on the pegvaliase prescribing information regarding the risk of low maternal blood phe, we hypothesized that pegvaliase treatment would not alter the phenylalanine content of the treated mother's breast milk. As shown above, we did not detect any difference in breast milk phenylalanine content regardless of whether the mother was treated with pegvaliase or not.

Another consideration in allowing the mother to breastfeed while on pegvaliase was to prevent the adverse neurocognitive, nutritional, and psychological consequences for the mother that are associated with maintaining a Phe-restricted diet [15], especially while caring for an infant [16]. The metabolic team and mother thought it best to for her maintain the Phe-restricted diet during the first two weeks of lactation to avoid the additional stress of restarting pegvaliase while establishing breast-feeding and maternal bonding. Once pegvaliase was restarted, the dose escalation and the return to a normal diet proceeded uneventfully. While the post-partum plan was to monitor blood Phe once to twice weekly, once the mother's blood Phe had stabilized, fewer samples were received, illustrating that even in a motivated patient the difficulty of blood Phe monitoring should not be minimized.

The offspring's growth and development have been normal. Relative weaknesses in early language development were noted in maternal PKU offspring during the preschool years [17], and may have persisted through age 7 years even in children whose mothers were treated prior to pregnancy [18]. However, as in this little boy's case, scores were well within the average range.

Evidence about the safety of pegvaliase during pregnancy and lactation is scarce [19], and our clinic's judgement about its use during this time has been cautious and patient-dependent. For our patient who previously had exhibited the ability to return to a phenylalanine-restricted diet and maintain good metabolic control for pregnancy, we concluded that it was safest for her to discontinue pegvaliase again for her second pregnancy; whereas for another patient who was incapable of maintaining the diet, the risk to benefit ratio may be different. Also, after our patient's previous pregnancy, pegvaliase was not prescribed during lactation, but evidence gained from that experience suggested that pegvaliase therapy during lactation would be safe, and that the nutritional and psychological cost of maintaining the diet during lactation was not necessary. The reinstitution of pegvaliase therapy occurred over a 4-week period to reach the woman's pre-pregnancy dose to ensure that she did not have hypersensitivity reactions and to allow a transition time back to a normal diet. However, during the pegvaliase clinical trials, study participants in the placebo group restarted the drug after an 8-week withdrawal period at their previous assigned dose of 20 or 40 mg/day without reported adverse events [5]. In future situations where a woman plans to breastfeed, we would likely increase the dose over a 4-week period again, but the approach taken would depend on the patient's pre-pregnancy pegvaliase dose and history of hypersensitvity reactions.

## 4 Conclusion

The absence of pegvaliase activity and the normal Phe content of the breast milk samples, as well as the infant's normal growth and development, indicate that pegvaliase therapy during lactation is safe.

Further studies would be helpful to confirm this; however, it appears that a woman with PKU, if she so chooses, can successfully breastfeed her infant while also having the benefit of excellent blood Phe control on pegvaliase. This, in turn, can also positively impact the infant's health and development.

## Funding source

This research did not receive any specific grant from funding agencies in the public, commercial, or not-for-profit sectors.

## Data Availability

No data was used for the research described in the article.
